# Metaphoric medicine: A retrospective clinical audit of glycaemic outcomes associated with an analogy-based consultation framework for insulin-treated type 2 diabetes mellitus

**DOI:** 10.51866/oa.1119

**Published:** 2026-05-24

**Authors:** Muhammad Hanif Omar, Zainal Fitri Zakaria, Wan Nusairi Wan Mohd Nasruddin, Muzawani Mokhlis

**Affiliations:** 1 Klinik Kesihatan Karak, Jalan Besar Karak, Bentong, Pahang, Malaysia.; 2 Klinik Kesihatan Batu Rakit, Kampung Mengabang Telung, Kuala Nerus, Terengganu, Malaysia.; 3 Klinik Kesihatan Bekok, Segamat, Johor, Malaysia.; 4 Klinik Kesihatan Senawang, Seremban, Negeri Sembilan, Malaysia.

**Keywords:** Type 2 diabetes mellitus, Insulin, Patient education as topic, Quality improvement, Metaphor

## Abstract

**Introduction:**

Poor glycaemic control among patients with insulin-treated type 2 diabetes mellitus (T2DM) in Malaysia remains a major challenge despite increased insulin use. Patient education is limited by complex medical explanations and lack of standardised consultation protocols. Methods: We conducted a retrospective clinical audit of patients with T2DM attending a structured self-monitoring of blood glucose (SMBG) clinic from January to July 2023. Patients on insulin with glycated haemoglobin (HbA1c) levels of >8.5% were consecutively enrolled into a multicomponent care package comprising the Metaphoric Medicine Practical Roadmap for Insulin Management Essentials (M2-PRIME) consultation framework, an analogy-based educational tool employing the ‘garbage and lorry’ metaphor as well as structured 6-week follow-ups, SMBG review and insulin dose adjustment. The primary outcomes were changes in HbA1c and fasting blood sugar (FBS) levels at 6 months.

**Results:**

Eighty-nine patients completed follow-up (mean age=57.2 years; women=57.5%). The mean baseline HbA1c and FBS levels were 11.88% (±1.84) and 12.54 mmol/L (±6.32), respectively. After 6 months, the HbA1c level decreased by 1.57% (95% CI=1.14, 2.00; P<0.001) and the FBS level by 3.32 mmol/L (95% CI=2.21, 4.44; P<0.001). The HbA1c and FBS levels improved in 63 (70.8%) and 59 (66.3%) patients, respectively.

**Conclusion:**

Consultations incorporating the M2-PRIME were associated with clinically meaningful pre-post reductions in HbA1c and FBS levels among poorly controlled insulin-treated T2DM cases. However, without a control group, the independent contribution of the M2-PRIME cannot be isolated from concurrent insulin intensification and structured follow-up. These findings are preliminary and hypothesis-generating. A prospective randomised controlled trial is warranted.

## Introduction

The burden of type 2 diabetes mellitus (T2DM) on Malaysians continues to grow, as evidenced by the latest 2023 National Health and Morbidity Survey. The findings highlight a concerning prevalence rate of 15.6%, placing increasing strain on the public healthcare system.^1^

While there are newer effective treatment modalities for T2DM, the utilisation of insulin therapy among patients with T2DM in Malaysia remains high and has been on the rise to align with local clinical practice guidelines.^2^ Despite this, only 14.2% of patients with T2DM on insulin therapy achieved good glycaemic control according to the National Diabetes Registry Report 2013–2019.^3^

An often-neglected barrier to optimal glucose control is patients’ comprehension and knowledge of diabetes itself. Multiple Malaysian studies have highlighted the lack of patient education on how to optimise insulin therapy.^4,5^ In addition, past research has found a correlation between T2DM selfmanagement education and improved glycaemic control.^6^

In Malaysia’s government health clinic settings, primary healthcare practitioners (PHPs) play a crucial role in providing education to patients with T2DM. With the shift to personalised care, also referred to as patient-centred approach, which is currently advocated worldwide,^7,8^ novel approaches that are cost-effective and widely applicable are much needed in local settings. To the authors’ knowledge, there are currently no local guidelines on how to consult patients with T2DM in Malaysia.

To address this gap in standardised, patient-centred consultation, we devised the Metaphoric Medicine Practical Roadmap for Insulin Management Essentials (M2-PRIME), a consultation module tailored for primary care physicians and intended for patients with T2DM on insulin. This module incorporates a comprehensive, patient-centred consultation framework based on local clinical practice guidelines. It encompasses rapport-building, history-taking, diet and exercise advice and insulin use and intensification, employing the ‘garbage and lorry’ analogy as a unifying thread for each topic addressed during the consultation, assisted by visual aids.

This retrospective clinical audit aimed to explore the glycaemic outcomes associated with the developer-delivered M2-PRIME consultation framework. The M2-PRIME was evaluated as the specific educational component within a broader, multi-component care package that additionally incorporated structured follow-up and active insulin dose adjustments to clearly distinguish the intervention from routine clinical care. This integrated approach was implemented in a specialised self-monitoring of blood glucose (SMBG) clinic, with preliminary fasting blood sugar (FBS) and HbA1c levels serving as the primary outcome measures.

## Methods

### Overview

This retrospective clinical audit and quality improvement study was conducted at the SMBG clinic, a sub-clinic under Klinik Kesihatan Senawang, Malaysia, from January to July 2023. The clinic operates once weekly and is managed by two PHPs: a family medicine specialist trainee and a medical officer, who were both the primary developers of the M2-PRIME framework. Because this was a developer-delivered quality improvement initiative, formal external training was not required to administer the framework.

The framework was developed with sample consultation dialogues to encourage personalisation, adaptability and modifiability during patient encounters. However, to ensure intervention fidelity, internal consistency and future reproducibility, both PHPs adhered to the core pre-defined consultation scripts and utilised standardised physical visual aids for the ‘garbage and lorry’ analogy during every patient encounter.

Participants received standard diabetes care through the SMBG clinic instead of the usual non- communicable disease clinic. This study involved a retrospective review of existing clinical records of patients who had already received standard care under the SMBG service. No additional procedures, randomisation or patient contact was conducted for research purposes.

The M2-PRIME consultation framework had been introduced as part of a routine clinical service improvement initiative, not as an experimental intervention. All data were anonymised before extraction, and no identifiable patient information was used in the analysis.

### M2-PRIME consultation framework

The M2-PRIME consultation framework integrates core elements of the Malaysian T2DM clinical practice guidelines into a structured consultation guide. It includes rapport-building, medical and lifestyle history-taking, dietary and exercise counselling, insulin education and dose intensification where appropriate.

The M2-PRIME aims to simplify complex concepts ofT2DM, making them accessible to patients regardless of their educational background. To achieve this, it employs the ‘garbage and lorry’ analogy as a unifying metaphor to explain diabetes pathophysiology and insulin action. Visual aids and simplified language are used to promote comprehension across varying literacy levels.

### SMBG clinic

The SMBG clinic is a specialised, once-weekly service managed by the two PHPs who developed the M2-PRIME framework. Consecutive enrolment included all individuals attending the clinic on active insulin therapy with an HbA1c level of >8.5%. In summary, enrolled participants received a structured, multi-component diabetes care that included the following: (1) M2-PRIME analogy- based educational consultation; (2) structured follow-up at 6-week intervals; (3) review of selfmonitored blood glucose records; and (4) insulin dose and regimen adjustment guided by clinical response. The final analysed cohort consisted strictly of all individuals who completed the full protocol of exactly three M2-PRIME sessions and underwent repeat HbA1c testing approximately 2 months after their final visit, regardless of their final glycaemic outcome.

Successful reduction of a repeat HbA1c level to <8.5% resulted in formal discharge from the clinic. Those who remained at >8.5% continued their regular 6-week follow-ups; however, any data beyond this initial 6-month assessment period were not extracted for this study. Conversely, defaulting on two consecutive appointments led to discontinuation from the service.

### Inclusion and exclusion criteria

Patients were eligible for inclusion when they were established patients with T2DM already on insulin therapy (regardless of insulin type, regimen or treatment duration), had an HbA1c level greater than 8.5% at referral and had previously been seen by a diabetes educator or dietitian prior to referral. The sole exclusion criterion was current or newly confirmed pregnancy during the follow-up period.

### Engagement

During the first visit, participants were clerked regarding their T2DM using the M2-PRIME framework to identify the reason behind their uncontrolled T2DM. Using standardised visual aids and script guide, the PHPs presented the M2-PRIME ‘garbage and lorry’ analogy to explain the T2DM disease processes, food and lifestyle advice and pharmacology of their current treatment.

In this analogy, blood sugar was framed as ‘garbage’ in the bloodstream, while insulin acted as the ‘lorry’ responsible for transporting this garbage to the body’s ‘factory’ (the cells) to be used for energy. The content of the consultation guide and analogies used in the M2-PRIME are summarised in Table 1.

**Table 1:** Summary of the consultation contents of the M2-PRIME.

Part	Title	Content	Goal
1	Re-clerking the patient	Social, co-morbidities, diabetes, diet, activities, insulin, monitoring history	Building rapport, assessing insight and understanding the patient’s potential causes of uncontrolled T2DM
2	‘Garbage, lorry and factory’	Explanation of the pathophysiology of T2DM using analogies	Understanding the reason of why T2DM happens
3	Reducing garbage	Explanation of how carbohydrates, protein and fibre affect blood sugar (garbage)	Making better dietary choices tailored to the patient’s lifestyle
4	Refurbishing the factory	Explanation of lifestyle changes that could improve insulin resistance	Understanding how active lifestyle can improve blood sugar
5	Optimising lorries	Explanation of the pharmacology of insulin, different types available and justification for each type	Understanding why patients need insulin and clearing up misconceptions
6	Personalisation and intensification	Starting an intensification protocol based on the patient’s goals	Self-empowerment to adjust insulin and SMBG education

T2DM: type 2 diabetes mellitus; M2-PRIME: Metaphoric Medicine Practical Roadmap for Insulin Management Essentials; SMBG: self-monitoring of blood glucose.

The consultations focused on patient empowerment on self-adjustment of patients’ insulin based on their SMBG readings. Participants were given an intensification protocol as outlined in the M2- PRIME framework, with a 6-week appointment to review their condition and SMBG readings. All diet, lifestyle and clinical guidance followed Malaysia’s T2DM clinical practice guidelines. The first consultation lasted up to 30 minutes, depending on the complexity of cases.

On subsequent appointments, the focus shifted to optimising patients’ insulin based on their lifestyle and diet modification, guided by their latest SMBG readings. Any issues or concerns were addressed during these sessions. These consultations lasted up to 15 minutes.

### Operational definitions

For the purposes of directional outcome analysis, ‘improved’ was defined as any numerical decrease in HbA1c or FBS levels from baseline to follow-up; ‘stable’ was defined as no numerical change; and ‘worsened’ was defined as any numerical increase. These definitions were intentionally inclusive of even small changes and were not considered to imply clinical significance.

### Outcomes

A total of 106 patients were enrolled in the structured SMBG clinic from 1 January 2023 to 1 July 2023. Among them, 17 (16.0%) were lost to follow-up and therefore excluded from the analysis. Data collected were entered into an abstraction form to be de-identified. Demographic data collected were age and sex, while clinical data collected were baseline HbA1c and FBS levels at the first and fourth visits (after 6 months) as well as baseline and final insulin regimens. The demographic and baseline clinical characteristics of the final sample (N=89) are presented in Table 2. The participants exhibited poor glycaemic control at baseline, with a mean HbA1c level of 11.88% (±1.84) and a mean FBS level of 12.54 mmol/L (±6.32).

**Table 2:** Demographic and clinical characteristics of the participants (N=89).

Characteristic	Value
Age (year)	Mean: 57.2±10.7 (range: 35-83)
Female sex	51 (57.3%)
Male sex	38 (42.7%)
Baseline HbA1c level (%)	11.88±1.84
Baseline FBS level (mmol/L)	12.54±6.32
Baseline insulin regimen	
– Basal-bolus	33 (37.1%)
– Pre-mixed	40 (44.9%)
– Basal only	15 (16.9%)
– Basal plus	1 (1.1%)

HbA1c = glycated haemoglobin; FBS = fasting blood sugar; SD = standard deviation.

Percentages were based on the total sample. HbA1c: glycated haemoglobin, FBS: fasting blood sugar. Outcomes were analysed for 89 patients who completed follow-up.

## Results

Paired-samples t-tests confirmed significant reductions in both glycaemic parameters following 6 months of three M2-PRIME-guided consultations (N=89). The HbA1c level decreased by a mean of 1.57% (95% CI=1.14, 2.00; t(88)=7.218, P<0.001), from a baseline of 11.88% (±1.84) to 10.31% (±2.30), with a large effect size (Cohen’s d=0.77, 95% CI=0.53, 1.00). Directional analysis indicated reductions in 70.8% (63/89) of the participants, stability in 19.1% (17/89) and increases in 10.1% (9/89). The FBS level decreased by a mean of 3.32 mmol/L (95% CI=2.21, 4.44; t(88)=5.928, P<0.001), from a baseline of 12.54 mmol/L (±6.32) to 9.22 mmol/L (±6.37), with a medium-to-large effect size (Cohen’s d=0.63, 95% CI=0.40, 0.85). Reductions occurred in 66.3% (59/89) of the participants, stability in 18.0% (16/89) and increases in 15.7% (14/89).

The outcomes of all 89 patients who completed follow-up were analysed. An independent-samples t-test was conducted, comparing the baseline HbA1c level of the 89 completers against that of the 17 participants lost to follow-up, to assess for potential attrition bias. There was no significant difference in the mean baseline HbA1c level between the two groups (11.88% vs 11.79%, P=0.852).

**Figure 1:**
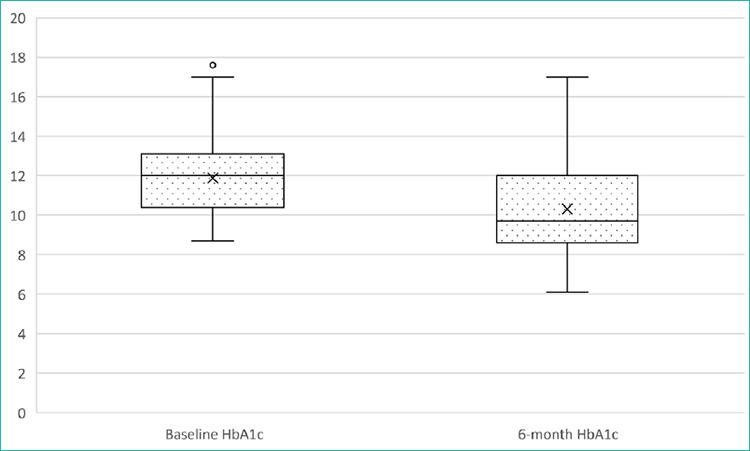
Box plot of the HbAlc levels before and after the M2-PRIME-based intervention. Boxes show the IQR with a median line; whiskees represent 1.5xIQR, end dofs indicate the outliers. The mean HbA1c level decieased significantly from baeeline to 6 months (P<0.001). IQR: inieequartile eange; HbA1c: giycated haemoglobin.

**Figure 2:**
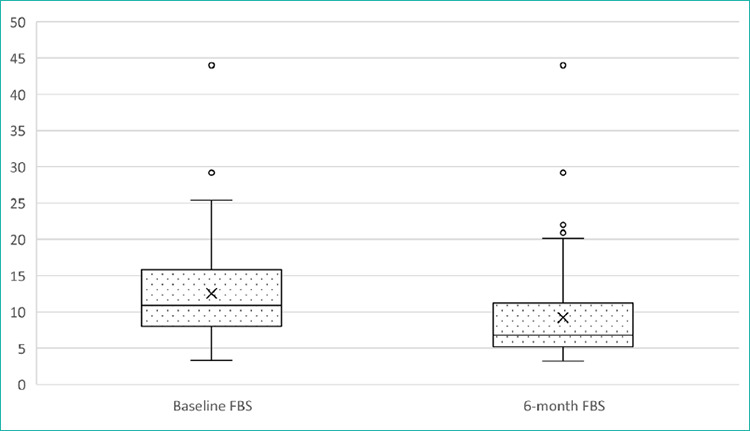
Box plot of the Fasting Blood Sugar (FBS) ievels befote and after the M2-PRIME- based intervention. Boxes show the IQR with a median line; whiskers represent 1.5xIQR, and dots indicate the outliers. The mean FBS level decreased significantly from baseline to 6 months (P<0.001). FBS: fasting blood sugar, IQR: interquartile range.

The glycaemic changes are illustrated in Figure 1 (HbA1c levels) and Figure 2 (FBS levels), while the inferential statistics and dnecgion ality of tlie changes are detailed in Table 3.

Lastly, as detailed in Table 2, the most common insulin regimen at baseline was pre-mixed insulin (44.9%, n=40), followed by basal-bolus (37.1%, n=33) and basal-only (16.9%, n=15) regimens. By the 6-month follow-up, a clinical shift towards more intensive therapy was observed; the proportion of patients utilising basal-bolus regimens increased to 40.4% (n=36), while the proportion of those on pre-mixed regimens decreased to 40.4% (n=36). Patient-level data on which individual patients underwent regimen escalation and whether their HbA1c change differed from those without escalation were not available from the retrospective clinical records. The exact insulin total daily dose (TDD) in units was also not systematically recorded.

**Table 3:** Clinical outcomes following the M2-PRIME-based intervention.

Outcome measure	Value
Baseline HbA1c level, mean ± SD	11.88±1.84
Follow-up HbA1c level, mean ± SD	10.31±2.30
Mean HbA1c change (95% CI)	-1.57 (-2.00, -1.14), P<0.001
Effect size for the HbA1c level (Cohen’s d)	0.77 (large)
Improved HbA1c level, n (%)	63 (70.8)
Stable HbA1c level, n (%)	17 (19.1)
Worsened HbA1c level, n (%)	9 (10.1)
Baseline FBS level, mean ± SD	12.54±6.32
Follow-up FBS level, mean ± SD	9.22±6.37
Mean FBS change (95% CI)	-3.32 (-4.44, -2.21), P<0.001
Effect size for the FBS level (Cohen’s d)	0.63 (medium-large)
Improved FBS level, n (%)	59 (66.3)
Stable FBS level, n (%)	16 (18.0)
Worsened FBS level, n (%)	14 (15.7)

Mean values are expressed as means ± SDs. HbA1c: glycated haemoglobin; FBS: fasting blood sugar; SD: standard deviation; CI: confidence interval.

## Discussion

This retrospective clinical audit explored the preliminary glycaemic outcomes associated with the developer-delivered M2-PRIME consultation framework, which was integrated as the specific educational component of a broader, multi-component care package within a specialised SMBG clinic. After 6 months, significant reductions were observed in the mean HbA1c (-1.57%) and FBS levels (-3.32 mmol/L), with a large proportion of participants (70.8% and 66.3%, respectively) showing improvements. While we acknowledge that the post-intervention mean HbA1c level of 10.31% is still above the recommended targets, these reductions are still clinically meaningful. The directionality analysis further supports the robustness of this effect, with more than two-thirds of the participants achieving substantial progress in glycaemic control. The categorical regimen data confirmed that insulin intensification occurred concurrently in a proportion of the patients. However, as individual patient-level regimen and dosing data were unavailable, the relative contributions of pharmacological intensification and the M2-PRIME educational component to the observed glycaemic changes cannot be determined.

In this study, 16.0% of the patients were lost to follow-up, which initially raises the possibility of selection bias. However, the attrition analysis revealed no significant difference in the baseline HbA1c level between the completers and non-completers (P=0.852). This suggests that the loss to follow-up was likely random and did not skew the baseline severity of the final analysed cohort.

Notably, the participants had already received standard primary care education and remained on active insulin therapy yet retained persistently poor glycaemic control, with a mean baseline HbA1c level of 11.88%. While regression to the mean remains a plausible explanation for part of the observed 1.57% reduction given this highly elevated baseline, the structured intensification of both education and pharmacotherapy may have jointly contributed to the improvement. In this context, the M2-PRIME functioned as a ‘rescue’ educational intervention where standard primary care counselling had previously been insufficient. However, without a concurrent control group, the independent contribution of this educational component cannot be established.

The HbA1 c reduction observed echoes the outcomes from structured diabetes education programmes. Teng et al. reported absolute HbA1c reductions ranging from 0.9% to 1.7% in pharmacy-led diabetes medication therapy adherence clinics.^9^ Importantly, both interventions achieved these results by combining structured patient education with active medication optimisation. In contrast, previous efforts of piloting new integrated care models such as Enhanced Primary Healthcare in Malaysia were found to be effective in improving the process of care but not the clinical outcomes, as HbA1c reductions were not statistically significant.^10^ Internationally, a systematic review of the effectiveness of nurse-led care for patients with diabetes^11^ revealed that HbA1c reductions varied but were most evident when nurses received formal training, used treatment algorithms and offered defined culturally sensitive and appropriate care.

From an implementation perspective, our findings resonate with those of the systematic review by Kovacs et al.,^12^ which found that single-component, interactive and locally adapted interventions were more effective in primary care guideline implementation than multifaceted or passive strategies. Cost-wise, the systematic review by Wan Rohimi and Mohd Tahir^13^ found that all types of educational interventions were highly likely to be cost-effective.

The M2-PRIME framework adheres to these principles: It is a low-cost, focused, metaphor-driven tool that actively engages patients, is culturally tailored to the Malaysian context and is feasible for delivery by PHPs in busy clinics. These design features likely contributed to its effectiveness in producing meaningful clinical outcomes in this study.

A key feature of the M2-PRIME is its use of metaphor: the ‘garbage and lorry’ analogy, supported by visual aids. Through various studies, analogies are recognised cognitive tools that simplify complex medical concepts, improve recall and enhance patient engagement.^14-16^ Their effectiveness has been well documented in other complex disease contexts,^15,17^ and our findings suggest similar benefits in T2DM care.

Mechanistically, the success of the M2-PRIME framework can be explained through the cognitive load theory.^18^ Standard diabetes education often involves complex physiological explanations and medical jargon, which impose a high extraneous cognitive load that easily overwhelms a patient’s working memory.^19^ By mapping complex medical concepts onto a highly familiar schema, the M2-PRIME drastically reduces extraneous cognitive load. This approach allows patients to process, retain and act upon clinical instructions using pre-existing mental models, thereby overcoming the psychological barriers and poor health literacy that typically hinder insulin adherence and titration.

### Limitations

First, and most critically, the absence of a control group means that the observed glycaemic improvements cannot be causally attributed to the M2-PRIME framework alone nor to any single component of the broader care package. In particular, structured follow-up and concurrent insulin intensification are major confounders that could independently account for the observed HbA1c and FBS reductions. Because this study was a retrospective audit of routine clinical records, exact insulin TDDs were not systematically recorded. Regimen-shift data serve only as a categorical proxy for intensification, precluding any adjusted or stratified analysis linking individual regimen changes to glycaemic outcomes. This represents the primary methodological limitation of this study. Additionally, given the markedly elevated baseline HbA1c level (11.88%), statistical regression to the mean must be acknowledged as a natural confounder to the observed improvements.

Second, restricting the final analysed cohort to patients who completed exactly three visits and underwent repeat HbA1c testing resulted in a 16.0% loss to follow-up. This introduces potential selection bias, as patients who defaulted may represent individuals with poorer engagement or worse clinical trajectories, which could lead to an overestimation of the observed benefit. Although the attrition analysis indicated no significant difference in the baseline HbA1c level between the completers and non-completers (11.88% vs 11.79%, P=0.852), this comparison was limited to the baseline values. Because the final 6-month glycaemic outcomes for the non-completers remain unknown, we cannot rule out differential outcomes at follow-up.

Third, only clinical outcomes were measured. The absence of psychosocial, behavioural and patient- reported outcome measures such as patient satisfaction, insulin adherence or diabetes knowledge acquisition restricts a more comprehensive evaluation of the educational intervention’s true impact; future studies must incorporate these metrics. Furthermore, the short 6-month observation period limits conclusions about the durability of any glycaemic improvements.

Lastly, as the study intervention was a developer-delivered intervention implemented at a single site, there is an inherent risk of performance bias. The developers’ deep familiarity and implicit expertise with the framework may have optimised its delivery, yielding uncertainty regarding scalability. Future multi-centre studies involving independent practitioners are required to test the true generalisability of the M2-PRIME.

## Conclusion

This retrospective audit provides preliminary, hypothesis-generating data indicating that the M2-PRIME consultation framework, delivered by PHPs within a structured SMBG clinic, was associated with clinically meaningful improvements in glycaemic control among patients with poorly controlled insulin-treated T2DM. However, the findings must be interpreted in the context of several critical limitations. Most notably, the lack of a control group and the unavailability of exact insulin dose data mean the independent effect of the M2-PRIME framework cannot be isolated from concurrent pharmacological intensification. Furthermore, the results are subject to potential regression to the mean, selection bias stemming from the strict three-visit completion requirement and a limited 6-month observation period. Finally, the absence of psychosocial outcome measures and the inherent performance bias of a single-site, developer-delivered implementation restrict broader generalisations. A prospective, multi-centre randomised controlled trial that incorporates patient-reported and behavioural outcomes is required to establish the independent efficacy of the M2-PRIME framework.

## Data sharing statement

The data and materials necessary to reproduce the findings reported in this manuscript are available at the Open Science Framework (doi:10.17605/0SF.I0/V26KU) immediately after publication without an end date. The M2-PRIME tool is also made available. Anyone can access the data, and the data can be used for any purpose.

**How does this paper make a difference in general practice?**

- The Metaphoric Medicine Practical Roadmap for Insulin Management Essentials (M2- PRIME) uses culturally relevant metaphors to translate complex type 2 diabetes concepts, bridging health literacy gaps.
- M2-PRIME integration in primary care may be associated with clinically meaningful reductions in glycated haemoglobin (HbA1c) and fasting blood sugar (FBS).
- By anchoring patient education to a standardised analogy, the M2-PRIME may be an effective approach to promote consistency in clinical communication.
- As a resource and cost-efficient tool, M2-PRIME may have high feasibility for delivery in busy primary care clinics.
- M2-PRIME’s analogy-based methodology shows strong potential for transferability to other chronic diseases.
